# Supplementary material for: Radiomics and gene expression profile to characterise the disease and predict outcome in patients with lung cancer
Source: Eur J Nucl Med Mol Imaging. 2021 May 7;48(11):3643–55. doi: 10.1007/s00259-021-05371-7 (PMC8440255; doi:10.1007/s00259-021-05371-7)
Supplement: Supplementary file 1 — (DOCX 1197 kb). [file 259_2021_5371_MOESM1_ESM.docx]

**Radiomics and gene expression profile to characterise the disease and predict outcome in patients with lung cancer**

Margarita Kirienko^1,5^, Martina Sollini^1,2^*, Marinella Corbetta^1^, Emanuele Voulaz^1,2^, Noemi Gozzi^2^, Matteo Interlenghi^3,4^, Francesca Gallivanone^3^, Isabella Castiglioni^3,6^, Rosanna Asselta^1,2^, Stefano Duga^1,3^, Giulia Soldà^1,2^*, Arturo Chiti^1,2^

1. Department of Biomedical Sciences, Humanitas University, Via Rita Levi Montalcini 4, 20090 Pieve Emanuele, Milan, Italy
2. IRCCS Humanitas Research Hospital, Via Manzoni 56, 20089 Rozzano – Milan, Italy
3. Institute of Molecular Bioimaging and Physiology, National Research Council (IBFM-CNR), Milan, Italy
4. DeepTrace Technologies s.r.l., via Conservatorio 17, 20122, Milan, Italy
5. Fondazione IRCCS Istituto Nazionale Tumori, Via G. Venezian 1, 20133 Milan, Italy
6. University of Milan-Bicocca, Department of Physics “G. Occhialini”, Piazza della Scienza 3, 20126, Milan, Italy

**ORCID (alphabetical order)**

Asselta: 0000-0001-5351-0619

Castiglioni: 0000-0001-7191-5417

Chiti: 0000-0002-5806-1856

Corbetta: 0000-0002-3774-7760

Duga: 0000-0003-3457-1410

Gallivanone: 0000-0001-6559-6157

Gozzi: 0000-0003-0126-7893

Interlenghi: 0000-0002-3066-4276

Kirienko: [0000-0002-3923-1151](https://orcid.org/0000-0002-3923-1151)

Soldà: 0000-0003-0972-610X

**Acknowledgement:**

We thank Daniela Pistillo, PhD for her support in accessing and managing biobank data. M. Sollini is supported by the Investigator Grant 2019-23959 funded by AIRC (Italian Association for Cancer Research) won by AC.

*Corresponding Authors

Martina Sollini, MD PhD

Department of Biomedical Sciences, Humanitas University, Via Rita Levi Montalcini 4, 20090 Pieve Emanuele – Milan, Italy

IRCCS Humanitas Research Hospital, Via Manzoni 56, 20089 Rozzano – Milan, Italy E-mail: martina.sollini@hunimed.eu

ORCID: 0000-0003-2214-6492

Giulia Soldà, PhD

Department of Biomedical Sciences, Humanitas University, Via Rita Levi Montalcini 4, 20090 Pieve Emanuele – Milan, Italy

IRCCS Humanitas Research Hospital, Via Manzoni 56, 20089 Rozzano – Milan, Italy

E-mail: giulia.solda@hunimed.eu

ORCID: 0000-0003-0972-610X

**ABSTRACT**

**Objectives** The objectives of our study were to assess the association of radiomic and genomic data with histology and patient outcome in non-small cell lung cancer (NSCLC).

**Methods** In this retrospective single-centre observational study, we selected 151 surgically treated patients with adenocarcinoma or squamous cell carcinoma who performed baseline [18F]FDG PET/CT. A subgroup of patients with cancer tissue samples at the Institutional Biobank (n=74/151) was included in the genomic analysis. Features were extracted from both PET and CT images using an in-house tool. The genomic analysis included detection of genetic variants, fusion transcripts, and gene expression. Generalised linear model (GLM) and machine learning (ML) algorithms were used to predict histology and tumour recurrence.

**Results** Standardised Uptake Value (SUV) and kurtosis (among the PET and CT radiomic features, respectively), and the expression of *TP63*, *EPHA10*, *FBN2*, and *IL1RAP* were associated with the histotype. No correlation was found between radiomic features/genomic data and relapse using GLM. The ML approach identified several radiomic/genomic rules to predict the histotype successfully. The ML approach showed a modest ability of PET radiomic features to predict relapse, while it identified a robust gene expression signature able to predict patient relapse correctly. The best-performing ML radiogenomic rule predicting the outcome resulted in an Area Under the Curve (AUC) of 0.87.

**Conclusions** Radiogenomic data may provide clinically relevant information in NSCLC patients regarding the histotype, aggressiveness, and progression. Gene expression analysis showed potential new biomarkers and targets valuable for patient management and treatment. The application of ML allows to increase the efficacy of radiogenomic analysis and provide novel insights into cancer biology.

**Keywords**: Artificial intelligence, Image analysis, Lung cancer, Radiogenomics, PET/CT, mutation, gene expression.

**INTRODUCTION**

Lung cancer is a leading global cause of cancer-related deaths, with more than 2.2 million people diagnosed and 1.9 million deaths documented worldwide in 2017 [1]. The five-year survival rate is less than 25% when diagnosis occurs at a locally advanced or metastatic disease stage. The survival rate rises above 50% if the disease is diagnosed early when local treatment is feasible [2]. In unresectable disease, systemic therapy with cytotoxic and targeted drugs are the main treatment options [3,4]. Targeted therapy requires tumour tissue molecular profiling to identify specific biomarkers to tailor treatments [5]. Cancer cells are characterised by high genomic instability, responsible for accumulating somatic mutations in crucial oncogenic/oncosuppressor genes, driving uncontrolled cancer cell proliferation [6]. Genetic information may be used to predict survival, as a prognostic biomarker or response to treatment, as a predictive biomarker to support clinical decisions [7]. In particular, in lung cancer, genetic mutations in *ALK*, *BRAF*, *EGFR*, and *ROS1* guide treatment decisions in patients affected by advanced disease and recurrence [3,4]. Beyond these alterations, other oncogenic driver mutations - even if currently not targetable - include *RET*, *HER2*, *KRAS*, and *MET* [8]. Molecular data are not routinely used in early-stage lung cancer.

In cancer patients, features of tumours identified from imaging data (e.g., CT and PET) can be used as biomarkers to reveal diagnostic, predictive, and prognostic associations, based on the identification of correlations with pathological or molecular reference, response to treatment, or survival outcomes: this process is defined radiomics. Within this framework, image features extracted and used as predictors include lesion volume, shape, and texture descriptors [9]. Radiogenomics refers to the integration of imaging-derived parameters and genomic data to find clinically relevant associations. Imaging-based typing has the advantage that it can capture information from the whole tumour lesion, can be performed at multiple time points for treatment monitoring, and can be carried out when a biopsy is not feasible. It is cost-effective, relying on routinely acquired clinical imaging.

Imaging-genomic maps have been shown promising in predicting molecular alterations [10]. Additionally, the integration of data generated from complementary "omics" sources may improve single-domain predictive models [11]. Multi-dimensional omics datasets (i.e. large number of features per each subject) – especially when the biological/clinical significance of features is unknown [12] – have driven research towards the application of machine learning (ML) algorithms for analysis [13]. On one hand, ML is hampered by several issues including the “black-box” problem, trustworthiness, ethics, and responsibility [14–16]. However, it bears the advantage of learning directly from data and improving the prediction process [17]. Therefore, it is no wonder the close connection between radiomics and ML.

Current data supporting the efficacy of radiomics in lung cancer predicting diagnosis, prognosis, and optimal therapy are ample and promising and supports a future role for computer-assisted diagnosis and management in clinical oncology [12]. Nonetheless, radiogenomics in lung cancer patients is still in its early stages, and extensive data studies are needed to validate the concept [5]. Many radiomic and radiogenomic studies are burdened by limitations including models’ explainability and results’ interpretation. However, in some domains such as healthcare explaining and interpreting which features or how the artificial intelligence system is returning the predictions may be far more critical than model’s performance. Indeed, explainable and interpretable models may unveil information about biological pathways, chemical mechanisms, or neural substrates, potentially leading to new scientific insights [16,18,19].

The objectives of our study were 1) to assess the association of [18F]FDG PET/CT radiomic features with histology and patient outcome; 2) to identify gene expression alterations and mutations in early stage NSCLC and test their association with histology and patient outcome, and 3) to assess the association of radiogenomics with histology and patient outcome trough explainable methods (both traditional statistics and ML).

**METHODS**

*Study design*

In this retrospective single-centre observational study, we applied the following criteria to select patients from the Institutional database. Inclusion criteria were: i) age >18 years, ii) pathologic diagnosis of non-small cell lung cancer (NSCLC), iii) enrolment from November 2011 to April 2018; iv) availability of baseline [18F]FDG PET/CT, v) surgical treatment, and vi) availability of cancer tissue sample at the Institutional Biobank for those patients to be included in a subpopulation of the study cohort. Exclusion criteria were: i) diagnosis of other malignancies, but non-melanoma skin cancer, in the previous three years; ii) interval time between PET/CT and surgery >3 months; iii) neoadjuvant treatment; iv) NSCLC other than squamous cell carcinoma (SQC) and adenocarcinoma (AC) to avoid inhomogeneity within the patient cohort. We did not exclude patients with a history of non-melanoma skin cancers since they generally do not affect patients’ prognosis. The selection workflow is reported in Figure 1. Demographic parameters such as age and sex were collected for all patients. Smoking habits were recorded. Performance status was not considered in this analysis.

The institutional ethics committee approved the study (study number 1751). All the patients who donated their tissue samples to the biobank signed informed consent to use their data, imaging, and samples for research purposes; for the remaining patients, a specific, informed consent was waived because of the observational and retrospective study design.

*Image acquisition and processing*

[18F]FDG PET/CT image acquisition was performed according to versions 1.0 [20] and 2.0 [21] of the European Association of Nuclear Medicine (EANM) guidelines until and from February 2015, respectively. Briefly, patient preparation with fasting at least 4 hours before [18F]FDG injection and blood glucose levels below 200 mg/dl were requested. Images were acquired 60±5 min after injection of [18F]-FDG, using either a Siemens Biograph 6 LSO (Siemens, Erlangen, Germany) or a General Electric Discovery 690 (General Electric Healthcare, Waukesha, WI, USA) PET/CT scanner. All PET images were corrected for attenuation using the acquired CT data. Image acquisition parameters are reported in Supplementary Table 1.

The primary lung cancer lesions were delineated on PET images applying a fully automatic segmentation method, combining an automatic threshold-based algorithm to define the tumour volume and a k-means clustering algorithm to estimate the background [22]. CT target lesion was delineated using 3-D Slicer application FastGrowCut, implementing a competitive region growing algorithm using cellular automata [23]. The images were resampled to have isometric voxels with a 2 mm length. Calculation of PET parameters was performed using an in-house image processing tool, running on MATLAB based on the radiomic model of Vallières et al [24] and providing 60 features [25,26]. The partial volume effect correction was used for Standardized Uptake Value (SUV) calculation. CT radiomic features (n=57) extraction was performed using the HeterogeneityCAD tool implemented in the 3-D Slicer, according to Aerts et al. [27]. The different number of radiomic features for PET and CT resulted from the use of the two different processing tools. Image processing and calculation of image-derived parameters are reported in Supplementary Table 1, according to recommendations of the imaging biomarker standardisation initiative (IBSI) reporting guidelines [28].

*Pathology*

Histological type and staging classification were assessed according to good clinical practice on pathology samples obtained at surgery (AJCC manual). Fresh-frozen samples were collected and stored according to the Biological Biobank's Institutional procedures for those patients who donated their tissue.

*Molecular analyses (mutations and gene expression)*

Molecular analysis of 74 tumour samples (21 SQC, 53 AC) was performed using a targeted RNAseq approach. Besides, six normal tissue samples were evaluated as reference. Molecular analyses by targeted RNAseq included: 1) detection of genetic variants (both single nucleotide variants, SNVs, and small insertions/deletions, indels); 2) detection of fusion transcripts; 3) gene expression analysis. RNA extraction from fresh-frozen lung tissues, preserved in RNALater-ICE (Thermo Fisher Scientific, Waltham, MA, USA), was performed using either an automated procedure with the Maxwell RSC miRNA Tissue kit (Promega, Madison, WI, USA) or a standard protocol using the Eurogold TriFast reagent (Euroclone, Wetherby, UK). RNA quality was assessed on an Agilent 4200 TapeStation (Agilent Technologies; Santa Clara, CA, USA), obtaining a mean RNA integrity number (RIN) of 6.7 (max: 9, min: 4). Libraries were prepared starting from 55 ng of total RNA with the TruSight RNA Pan-Cancer Panel (Illumina, San Diego, CA, USA), following the manufacturer's instructions. This panel allows the simultaneous detection of fusion transcripts, point mutations, and gene expression changes, and it is characterised by a broad dynamic range, which can robustly detect RNAs of low abundance; it covers a total of 1,385 genes, including all major ones found mutated in lung cancer. Sequencing (76-bp paired-end reads) was performed on a NextSeq500 platform (Illumina). Data were analysed using the RNAseq alignment v. 2.0.10 pipeline on BaseSpace (Illumina). Briefly, input reads were filtered against abundant sequences, such as mitochondrial or ribosomal sequences, using Bowtie 0.12.9, and then aligned to the reference human genome (UCSC hg19) and the RefSeq annotation of transcripts with the Spliced Transcripts Alignment to a Reference (STAR) program (v. 2.6.1a). SNVs were identified with the Strelka Variant Caller v.2.9.9, and the presence of fusion transcripts was detected with the Manta Structural Variant Caller v.1.4.0. Gene/transcript expression was quantified by Salmon v.0.11.2. Differential expression analysis among histotypes (SQC, AC, and normal tissue) was evaluated with a likelihood ratio test (LRT) for significance using the DeSeq2 Bioconductor package [29]. We looked for known and possibly recurrent oncogenic variants to extract meaningful information to correlate with imaging and clinical data. Therefore, we selected variants with the following features: SNV, nonsynonymous (missense, nonsense, splice variants mapping at +/-2 position of the splice sites), and annotated in the Clinvar database (https://www.ncbi.nlm.nih.gov/clinvar/) as pathogenic or likely pathogenic. Concerning indels, we selected rare frameshift variants occurring only in cancer samples, covered by at least 10 reads, and with a Combined Annotation Dependent Depletion, CADD, score >15 [30].

*Follow-up and outcome assessment*

After surgery, further treatment and follow-up were performed according to standard procedures and guidelines after discussion at the multidisciplinary lung tumour board. As for outcome prediction, the endpoints of this study were disease recurrence, disease-free survival (DFS), and overall survival (OS). Disease recurrence was defined as relapse occurrence during follow-up. DFS was defined as the time between the date of surgery and either the date of recurrence or tumour-related death (event) or the date of last patient access (censored). OS was defined as the time between surgery and death (event) or last patient access date (censored).

*Statistical analyses*

All statistical procedures were carried out using specific R program packages, release 3.6.1 (http://www.r-project.org/). All P values were two-sided. P values of <0.05 were considered statistically significant.

*Conventional statistics*

Patient characteristics were summarised in frequency tables, and descriptive statistics were provided in terms of basic measures of central tendency (mean, median and range) and count proportion for continuous and dichotomic variables, respectively. Furthermore, correlation between age and sex either with histotype and tumour recurrence was tested. Lastly, the correlation between the two outcomes was analysed. For *KRAS/TP53/EGFR* mutations, carriers frequency data were performed using the Fisher exact test. Principal component analysis (PCA) was used to explore data; the first two components were used to visualize a possible clustering.

The Kaplan–Meier method was used to generate survival curves for the subgroups in each dataset, and the log-rank test was used to determine the statistical significance of differences (survminer R package).

For data analysis, we first performed an unsupervised clustering, using the pheatmap R package on log-transformed data, evaluating whether patients are distinguishable in the feature space (either by histotype or the tumour recurrence). After that, we used variables to fit a Binomial Generalised Linear Model (GLM) with logistic cumulative distribution function after checking for the correct normal distribution of residuals and the homogeneity of variance across the fitted values of the model. We used either the histotype (SQC or AC) or the tumour recurrence (yes or no) as the output variable. More specifically, for gene expression analysis, differentially expressed genes were selected using a false discovery rate (FDR) ≤0.001, a fold change ≥2, and a minimum average expression (baseMean) of at least 50 counts. Differential expression analyses identified genes specifically altered in cancer status compared to normal tissues and genes specifically altered in each histotype (SQC and AC). Differential expression analysis to identify transcriptional signatures associated with tumour recurrence (yes or not) was evaluated with a Wald test for significance and the DeSeq2 package. In this case, differentially expressed genes were selected using an FDR ≤0.05 and no threshold for fold change.

For radiogenomic analysis, we focused on the top differentially expressed genes, setting a stringent threshold for significance of FDR ≤0.001, fold change ≥2, and average expression (baseMean) among samples of at least 50 counts.

*Machine learning analysis*

In addition to classical statistics, we applied the Forecast environment of the Rulex (RULe eXtractor) 4.0 suite (www.rulex-inc.com), an integrated suite for the analysis through statistical/ML approaches. Rulex can manage all data types, including categorical/continuous variables, variables showing a high degree of correlation, or characterized by any data distribution.

Three datasets were used for analyses: 1) a dataset including only complete radiomic data (149 patients; two additional patients were not included because they were missing either PET or CT data); 2) a dataset using only molecular data (gene expression and *KRAS/TP53/EGFR* mutational status), comprising 74 patients; and 3) a dataset including only those 73 patients for which both radiomic and molecular data were available.

We applied the Logic Learning Machine (LLM) algorithm to our datasets using as an output variable either the histotype (SQC or AC) or the patient outcome (tumour recurrence yes or no). Each dataset was split into a training cohort and a test set (70% and 30%, respectively). Rulex LLM takes as input the features and returns a series of "rules" characterised by *n* conditions. As typical for decision rules algorithms [31], rule’s performance was expressed as coverage rate and error. The covering is the percentage of training patterns whose output value is equal to the rule's output that satisfy the rule (true positive); while the error is the percentage of the training patterns whose output value is different from the output of the rule that satisfy the rule (false positive). For each rule, accuracy and F1 were calculated.

We used the radiogenomic rule (integrating gene expression and radiomic data), which best-predicted tumour recurrence to build a score, and we calculated the corresponding receiving operating characteristic (ROC) curve and the area under the curve (AUC). For each condition, the score corresponded to the sum of 1 or 0 points.

**RESULTS**

Overall, 151 patients were included. Patient characteristics are reported in Table 1: 70% of patients developed an AC, 30% an SQC; the ratio between those that relapsed versus those that did not was exactly 1:1.

No correlations were observed between patients' age and the relapse status's histotype (P=0.078 and P=0.538, respectively). As for sex, a weak correlation was observed with histotype (being the male sex more common among SQC, with a male: female ratio=3.5, P=0.016). Instead, no correlation was evident between sex and relapse. Finally, we observed a weak correlation between the histotype and the relapse status, with AC cases more prone to relapse than SQC patients (P=0.032; the significance was also retained after correction for age, sex, and smoking status, P=0.026) (Supplementary Figure 1).

The two histotype patient groups did not show any significant difference in the overall survival rate nor their tendency to relapse (Supplementary Figure 2).

Mutation screening by targeted RNA sequencing identified more than 130,500 variants in the 80 analysed samples (74 tumours + 6 normal), with a mean of 1,632 variants per sample (1,577 SNVs, 27 deletions and 28 insertions). Each variant was covered on average by 162 reads (min: 3, max: 12,592). We obtained a list of the 142 topmost pathogenic variants, although very few of them were present in more than one sample. We found a missense *KRAS* mutation at codon 12 in 28% (21/74) of tumour samples and a *TP53* mutation in 55% (41/74) of cases (Supplementary Figure 3).

No known pathogenic variants or hotspot mutations were detected in *EGFR*, although eight samples carried rare nonsynonymous SNVs of uncertain significance (Supplementary Figure 4). Based on the variant type, location within the gene/protein, and predicted deleteriousness (evaluated by CADD, score >15), at least 6 (75%) of them might represent a likely pathogenic mutation. No recurrent gene fusions in the *ALK*, *ROS,* and *RET1* were detected.

Gene expression analysis on RNAseq data on 80 samples (21 SQC, 53 AC, and 6 non-tumoral tissues) showed a prevalent clusterisation based on histotype at PCA, with normal tissues separating from tumour samples (Supplementary Figure 5). According to relapse status, no clusterisation was observed at least when considering the first two principal components (data not shown).

***1. Association of PET/CT radiomic data with histology and outcome***

A total of 60 PET and 57 CT radiomic features were extracted in 149 patients (see Table 1).

Unsupervised hierarchical clustering, based on log-transformed data of extracted radiomic features, showed a good clusterisation based on histotype (Supplementary Figure 6). Conversely, the same analysis did not perform well when discriminating patients based on relapse (data not shown).

PET and CT features were further independently analysed using GLM. GLM showed that among the 117 analysed features, two outperformed in discriminating AC vs SQC patients. SUV and kurtosis resulted in the best PET, and CT features, respectively, in predicting histology (Figure 2A). SUV resulted higher in SQC than in AC (16.91±7.92 and 10.13±5.77, respectively; P=2.89*10^-6^). Similarly, CT-derived kurtosis was greater in SQC than in AC (11.97±11.17 and 3.81±5.85, respectively; P=5.49*10^-6^). Notably, both variables survived Bonferroni corrections for multiple testing (threshold corresponding to P=0.00083 and P=0.00088 for the SUV and the kurtosis, respectively). Conversely, PET and CT feature poorly correlated with the relapse status. Indeed, the best performing PET and CT features in discriminating tumour recurrence were Kurtosis (P=0.035) and LRE (P=0.0096), respectively, but neither kurtosis nor LRE survived correction for multiple testing (Figure 2B).

The Rulex LLM software, comprehensively analysing all the 117 radiomic features, identified four and two rules to predict histology and tumour recurrence, respectively (Supplementary Table 2). Rule number 4 - composed of 8 different conditions, three based on PET and five based on CT features - was the most interesting (Table 2), reaching coverage of 85.7% with an error rate of 3.6% (accuracy = 93%). Rule number 1 composed of 6 PET-based conditions reached an accuracy of 81% in predicting tumour recurrence (Table 2).

***2. Association of genomic data with histology and outcome***

Mutation and gene expression data on the 74 cases were used to search for possible correlations with histotype and the relapse status. Genes specifically altered by relapse were investigated on the 68 samples with available data (31 relapses, 37 no relapse). Only four genes resulted differentially expressed with an FDR ≤0.05. Mutation analysis focused on the two genes most commonly mutated in our cohort (*KRAS*, *TP53*) and the *EGFR* gene, i.e. the sole - among genes guiding treatment decision in lung cancer patients [3,4] - being mutated in the analysed cases. Concerning histotype, we evidenced a profound difference in the frequency of *KRAS* mutation carriers between AC and SQC (37.7% and 4.8%, respectively; P=0.0040) (Table 3A). We also observed a significant association between *KRAS*/*EGFR* mutations and relapse status, not retained after correction for histotype (values between parentheses in Table 3B). Gene expression analysis focused on the 238 genes that resulted differentially expressed (at FDR ≤0.001 and a fold change ≥2) according to different tissue samples (normal tissue, AC, SQC; 187 genes), tumour histotypes (47 genes), and recurrent status (4 genes). Among these 238 genes, *TP63*, *FBN2*, *EPHA10*, and *IL1RAP* emerged as strongly associated with the histotype (P<1.5*10^-4^; all genes survive the Bonferroni corrections for multiple tests) (Figure 3).

None among the analyzed genes was associated below the Bonferroni threshold (P=0.00021) with the relapse status. The comprehensive analysis performed by the Rulex LLM approach focused on all the 238 genes, and the data on *KRAS*/*TP53*/*EGFR* mutational status are summarised in Supplementary Table 3. Rulex LLM proved to be very powerful in predicting the histotype (rule number 1 reached a coverage =94.3% with 95.9% accuracy) and, above all, in predicting the outcome (rule number 4 reached a coverage = 91.7% with 95.6% accuracy). In all cases, no conditions related to the mutational status emerged. The best-performing rule predicting the histotype was based only on two conditions (one related to expression levels of *TP63*), whereas the best rule predicting relapse stem from the expression levels of 5 different genes (*AURKA*, *HIST1H2AM*, *IL12Rb2*, *CXXC4*, and *RYR3*) as detailed in Table 2.

***3. Association of radiogenomic with histology and outcome***

Finally, we used the Rulex LLM approach for analyzing the 73 cases having the entire set of variables available (i.e. all PET and CT features) and data on *KRAS*/*TP53*/*EGFR* mutational status and on 238 differentially expressed genes).

Results of radiogenomics analysis are detailed in Supplementary Table 4. Interestingly, using histotype as an output variable results almost entirely overlapping with those already obtained for the predictions based on genomic data (Supplementary Table 2A). The slightly different covering values depended on the missing sample.

The best-performing rule in predicting the outcome (covering rate =73.3% with 88.2% accuracy) combined conditions based on gene expression data and a PET-derived feature (i.e. LRHGE) as detailed in Table 2. This rule's ROC curve resulted in an area under the curve of 0.87 (Figure 4).

**DISCUSSION**

Our study explored the ability of radiomics, genomics, and radiogenomics to provide clinically relevant information in lung cancer patients using explainable models. Indeed, both the Binomial Generalized Linear Model and the Rulex/LLM are recognized as interpretable approaches [16]. Rulex/LLM may be used with different complementary purposes including automatic data discovery, model building, and self-explanatory predictions. Specifically, the LLM produces conditional logic-base predictive models with the advantage to be fully explainable (https://www.rulex.ai/rulex-algorithms/). We did not find any radiomic feature, mutational status, or gene expression profile to predict relapse *per se* using conventional statistics. However, radiomic features and gene expression profile resulted to predict tumour recurrence when introduced in decision rules identified using ML. Specifically, the use of Rulex/LLM allowed: i) to confirm the central role of SUVmax in predicting histology which was empowered by the addition of radiomic parameters; ii) to identify a combination of relevant imaging-derived features in predicting outcome confirming the higher discriminative power of PET compared to CT-based biomarkers; and iii) to detect a gene expression pattern associated to lung cancer outcome. Interpretable models, revealing new insights on a disease, are an essential requirement for translating radiomics and artificial intelligence to clinical practice.

Additionally, the implementation of ML into clinical practice requires the integration of the radiomics and ML systems into applications that imagers and clinicians use in their routine practice. Radiomics workflow is time-consuming and requires additional software. Once robust evidence emerges, radiomics/ML models should be promptly implemented into clinical workstations to support everyday practice.

Gene expression data - analysed by Rulex LLM - proved to be very powerful both in predicting the histotype (rule number 1 reached a coverage =94.3% with 95.9 accuracy) and, above all, in predicting the outcome (rule number 4 reached a coverage =91.7% with 95.6 accuracy). The best-performing genomic rule in predicting relapse stem from the expression levels of *AURKA*, *HIST1H2AM*, *IL12Rb2*, *CXXC4*, and *RYR3*. The expression of *AURKA* - a gene that contributes to the regulation of cell cycle progression [32] - has been reported to be associated with poor prognosis in smoking-related lung AC [33]. Histones variants *HIST1H2*, *HIST1H3*, and *HIST1H4*, acting as transcriptional promoters or repressors of cancer-related genes, have been reported to be involved in tumour progression and metastasis [34]. In particular, in lung AC, *HIST1H2AM* has been counted among genes differentially expressed between smoking and non-smoking [35]. Our data suggested that the smoking habits induced changes in *AURKA* and *HIST1H2AM* genes, and their upregulation or downregulation might play a pivotal role in determining the outcome. Literature data supported the involvement of IL-12Rβ2 in tumour cell proliferation, apoptosis, and metastasis. The down-regulation of IL-12Rβ2 in lung AC seems to be a tumour escape mechanism [36], and the IL-12Rβ2 expression has been negatively associated with tumour progression [37]. The Disheveled (Dvl) inhibitor Idax, coded by the *CXXC4* gene [38], seems to be involved in tumour cell invasiveness and proliferation [39,40], and its expression is associated with poor prognosis [41]. Furthermore, *CXXC4*, being capable of inhibiting the mitogen-activated protein kinases (MAPK) signalling pathway [42], is emerging as a novel tumour suppressor [43]. Moreover, the downregulation of the MAPK signalling pathway reduces the expression of programmed cell death 1 ligand 1 (PD-L1) in lung AC cells [44]. The *RYR3* gene encodes for a ryanodine receptor which mediates the calcium release for many cellular processes. Ryanodine receptor has been reported to be involved in epithelial-mesenchymal transition, in tumour cell apoptosis and treatment resistance in some cancers, including lung AC [45–47]. These data may provide the rationale for post-operative risk stratification with a differential follow-up scheme. The patients operated on a more aggressive tumour may be closely investigated during follow-up to identify recurrence at an earlier time point. However, molecular testing to identify molecular biomarkers are currently performed on tumour samples collected from biopsies or cytological specimens; these are invasive procedures that are not always feasible, may result in inadequate sampling, and cannot characterise intra- and inter-tumour heterogeneity. Moreover, in the case of recurrence, repetition of a biopsy is not mandatory. Indeed, targeted therapies may be administered based on the molecular testing on the specimens obtained at diagnosis, assuming that no molecular modification arises between disease onset and recurrence [48]. Consequently, other methods to identify actionable biomarkers in NSCLC are emerging to address the need for complementing or replacing traditional testing on tissue and cytological samples.

We found that image-derived features were able to discriminate between NSCLC histotype (Figure 2). Similarly, several studies have successfully demonstrated an association between radiomic features and NSCLC tumour histology based on both CT and PET radiomic features. In the study by Wu et al. [49], 53 CT radiomic features from lung tumours of 350 patients were significantly associated with tumour histological subtype. Applying multivariate classifiers using radiomic features as input tumour histological subtype could be reliably predicted (AUC=0.72) [49]. While, Aerts et al. [27] reported a radiomic analysis of 440 features extracted from CT data of 1,019 patients affected by NSCLC and head-and-neck cancer. They found a significant association with histology (P=0.019, Chi-square test). While in the study by Koyasu et al., the authors found PET-based models to identify histological lung cancer subtype with an AUC up to 0.84 [50]. In a previous cohort of 534 patients with lung nodules, we have demonstrated radiomic features' ability to potentially classify primary lung cancer subtypes (AUC=0.59-0.70 for CT and =0.61-0.88 for PET)[51]. SUV appeared to be the best predictor in the present work, following published data [52], regardless of the statistical approach (i.e. conventional statistics and ML). Indeed, SUVmax was recognised by GLM as significant, and minimum SUV was included in the best radiomic rule to predict histology. Randomness due to high correlation and the inherent redundancy among SUVs parameters was probably at the basis of the model's selection of SUVmax instead of the minimum (selected by GLM and ML, respectively).

On the other hand, from the Rulex LLM analysis on radiogenomics, it emerged that gene expression data alone prevail on those coming from [18F]FDG PET/CT analyses in predicting histology. Interestingly, the best-performing rule in predicting the outcome (covering rate >73%) combined conditions based on gene expression data and PET-derived feature (i.e. LRHGE). Though this rule - less performing than the one found when analysing only gene expression data (Table 2) - it was noteworthy to underline that: i) the number of conditions for this rule was lower, ii) we "forced" the software to give priority to radiomic features (which indeed only came up for relapse predictions), and iii) the corresponding ROC curve gave an overall significant AUC of 0.87.

We identified several genes' levels of expression (e.g. *TP63* and *EPHA10*) to be associated with AC vs SQC. In particular, *TP63* overexpression, often due to gene amplification, is frequently found in SQC and has been associated with prolonged survival [53]. *EPHA10* (the ephrin receptor A10 belonging to the subfamily of receptor tyrosine kinases and involved in cell-cell communication, regulating cell attachment, shape, and mobility in neuronal and epithelial cells), primarily explored in breast cancers [54], has been recently described in lung cancer [55]. Its mutation has been newly described as associated with a favourable outcome in lung adenocarcinoma - but not in squamous cell lung cancer -, treated with immune checkpoint inhibitors [56]. This finding needs further investigation to characterise better this protein's role in lung cancer as a prognostic biomarker or potential therapeutic target.

For relapse prediction, PET and CT feature failed to predict tumour recurrence when analyzed with classical statistics. We found a couple of rules through the Rulex LLM approach to correctly prognosticate the outcome in a good percentage of cases (61-67% of covering with 78-81% accuracy). These findings underlined that a radiomic feature might efficiently differentiate tumour subtypes, but it is not sufficient to explain the complexity (i.e. biological phenotyping). Conversely, combining more radiomic features, enclosing several - complementary - information may summarize all those biological properties beyond the histotype that contributes to disease aggressiveness. Notably, the best-performing rule to predict tumour relapse comprised a total of 6 conditions all related to PET features. The inclusion of conditions related to CT-derived parameters determined a drop of performances, confirming literature data [57].

The study is limited by the retrospective design that determined using routinely acquired PET/CT images on two different scanners, but the number of images obtained using one scanner was well balanced compared to those acquired using the other one (52% versus 48%). Moreover, in our previous study, we demonstrated radiomics analysis's reliability since the predictive models performed in the same way when considering and not considering significantly different features among scanners [51]. The outcome has been evaluated in terms of occurrence or not of disease. This evaluation may have determined a modest prognostic ability. Future analyses will take into account time information related to recurrence and overall survival. We could not perform further analyses on independent tissue samples to confirm and deeply investigate the role of gene expression findings of our work because it was out of the scope. Future studies are planned to investigate the role of these genes as molecular biomarkers and targets.

In conclusion, the radiogenomic approach promises to extract relevant information regarding lung cancer histotype, aggressiveness, and progression. Gene expression may provide additional valuable information to guide patient management and follow-up. ML algorithms' application allows to increase the efficacy of transcriptomic and radiogenomic analysis and provides novel insights into cancer biology.

**Legends for Figures**

**Figure 1. Patients’ selection workflow (title).** Schematic representation of the patients’ selection workflow**.**

**Figure 2.** **Top PET and CT features discriminating patients based on their lung cancer histotype or their tendency to relapse (title).** The best discriminative features were identified using the generalised linear model approach to predict histology (A) and outcome (B). A) Boxplots show Standardised Uptake Value and Kurtosis, the best performing PET and CT features, respectively, in discriminating cancer histotype. B) Boxplots show kurtosis and LRE, the best performing PET and CT features, respectively, in discriminating tumour recurrence. Boxes define the interquartile range; thick central lines refer to the median. P values before Bonferroni correction are provided for each feature.

**Figure 3: Top differentially expressed genes discriminating patients based on their lung cancer histotype (title).**

The four box plots show mRNA expression levels of *TP63*, *FBN2*, *EPHA10*, and *IL1RAP* genes, with lung cancer individuals grouped upon histotype. Boxes define the interquartile range; thick central lines refer to the median. The P-value for the difference is indicated (t-test; the threshold for Bonferroni correction for multiple testing corresponding to P=0.00021).

**Figure 4. Clusterization of relapsing/non-relapsing patients based on the best-performing prediction rule evidenced by the Rulex LLM analysis (title)**. On the left: three-dimensional scatter plot of patients experiencing (blu dots) or not (red dots) relapse. Patients were plotted based on the three genes' expression levels evidenced by the Rulex LLM analysis (determining the first three conditions of rule number 4; see Table 2). On the right: ROC curve for differentiating relapsing and non-relapsing patients based on a “score” including the expression levels of the *CXXC4*, *PAK3*, and *GHR* genes, as well as on the radiomic parameter LRHGE_PET. For each patient, the score was built summing, for each of the four conditions of the rule (Table 2), 1 or 0 points. At the bottom rich corner of the ROC panel, the AUC value is reported.

***Declarations***

- **Funding**: The present project was supported by the grant won by AC (IG-2016-18585) funded by the AIRC (Italian Association for Cancer Research).
- **Authors' contributions**: AC, MK, and MS conceptualised the study; AC, SD, GS, RA, MK, and MS designed the study; MK screened patients; EV enrolled patients; MS and MK collected the clinical data; MK, FG, IC, and MI, performed image analysis; MC and GS performed the genomic analysis; RA performed data analysis; AC, SD, MS, MK, RA, GS and NG critically interpreted results; AC, RA, MK, MS, GS, and NG drafted the paper. AC coordinated and supervised the project's activities. All the authors critically revised the paper and approved the submitted version of the manuscript.
- **Availability of data and material**: The manuscript represents valid work, and neither this manuscript nor one with substantially similar content under the same authorship has been published or is being considered for publication elsewhere. Arturo Chiti had full access to all the data in the study and takes responsibility for the data integrity and the accuracy of the data analysis. Raw data are available on specific request to the corresponding author.
- **Code availability:** not applicable.

**Compliance with ethical standards:**

- **Consent to participate and consent for publication**: All the patients who donated their tissue samples to the biobank signed informed consent for the usage of their data, imaging and samples for a research purpose; for the remaining patients, because of the observational and retrospective study design, a specific, informed consent was waived.
- **Ethics approval:** The Ethics Committee of the Humanitas Clinical and Research Centre IRCCS approved the study on 18 April 2017, with the authorisation number 1751.
- **Conflict of interest**: Prof. Chiti reports a fellowship grant from Sanofi, personal fees from AAA, Blue Earth Diagnostics and General Electric Healthcare, outside the submitted work. The other authors do not report any conflict of interest.

**REFERENCES**

1. Global Burden of Disease Cancer Collaboration GB of DC, Fitzmaurice C, Abate D, Abbasi N, Abbastabar H, Abd-Allah F, et al. Global, Regional, and National Cancer Incidence, Mortality, Years of Life Lost, Years Lived With Disability, and Disability-Adjusted Life-Years for 29 Cancer Groups, 1990 to 2017: A Systematic Analysis for the Global Burden of Disease Study. JAMA Oncol. American Medical Association; 2019;

2. Goldstraw P, Chansky K, Crowley J, Rami-Porta R, Asamura H, Eberhardt WEE, et al. The IASLC Lung Cancer Staging Project: Proposals for Revision of the TNM Stage Groupings in the Forthcoming (Eighth) Edition of the TNM Classification for Lung Cancer. J Thorac Oncol. 2016;11:39–51.

3. Postmus PE, Kerr KM, Oudkerk M, Senan S, Waller DA, Vansteenkiste J, et al. Early and locally advanced non-small-cell lung cancer (NSCLC): ESMO Clinical Practice Guidelines for diagnosis, treatment and follow-up. Ann Oncol. 2017;28:iv1–21.

4. Planchard D, Popat S, Kerr K, Novello S, Smit EF, Faivre-Finn C, et al. Metastatic non-small cell lung cancer: ESMO Clinical Practice Guidelines for diagnosis, treatment and follow-up †.

5. Ninatti G, Kirienko M, Neri E, Sollini M, Chiti A. Imaging-Based Prediction of Molecular Therapy Targets in NSCLC by Radiogenomics and AI Approaches: A Systematic Review. Diagnostics (Basel, Switzerland). Diagnostics (Basel); 2020;10.

6. Hanahan D, Weinberg RA. Hallmarks of cancer: the next generation. Cell. 2011;144:646–74.

7. Berger MF, Mardis ER. The emerging clinical relevance of genomics in cancer medicine. Nat Rev Clin Oncol. 2018;15:353–65.

8. Kalemkerian GP, Narula N, Kennedy EB, Biermann WA, Donington J, Leighl NB, et al. Molecular testing guideline for the selection of patients with lung cancer for treatment with targeted tyrosine kinase inhibitors: American society of clinical oncology endorsement of the college of American pathologists/ international association for the. J Clin Oncol. 2018;36:911–9.

9. Sollini M, Cozzi L, Antunovic L, Chiti A, Kirienko M. PET Radiomics in NSCLC: State of the art and a proposal for harmonization of methodology. Sci Rep. 2017;7.

10. Jansen RW, van Amstel P, Martens RM, Kooi IE, Wesseling P, de Langen AJ, et al. Non-invasive tumor genotyping using radiogenomic biomarkers, a systematic review and oncology-wide pathway analysis. Oncotarget. 2018;9:20134–55.

11. Bodalal Z, Trebeschi S, Nguyen-Kim TDL, Schats W, Beets-Tan R. Radiogenomics: bridging imaging and genomics. Abdom Radiol. 2019;44:1960–84.

12. Sollini M, Antunovic L, Chiti A, Kirienko M. Towards clinical application of image mining: a systematic review on artificial intelligence and radiomics. Eur. J. Nucl. Med. Mol. Imaging. Springer; 2019. p. 2656–72.

13. Sollini M, Bartoli F, Marciano A, Zanca R, Slart RHJAJA, Erba PA. Artificial intelligence and hybrid imaging: the best match for personalized medicine in oncology. Eur J Hybrid Imaging. 2020;

14. Sollini M, Bandera F, Kirienko M. Quantitative imaging biomarkers in nuclear medicine: from SUV to image mining studies. Highlights from Annals of Nuclear Medicine 2018. Eur J Nucl Med Mol Imaging. 2019;

15. Sollini M, Cozzi L, Ninatti G, Antunovic L, Cavinato L, Chiti A, et al. PET/CT radiomics in breast cancer: Mind the step. Methods. 2020;

16. Guidotti R, Monreale A, Ruggieri S, Turini F, Giannotti F, Pedreschi D. A Survey of Methods for Explaining Black Box Models. ACM Comput Surv. ACM PUB27 New York, NY, USA ; 2019;51:1–42.

17. Kirienko M, Biroli M, Gelardi F, Seregni E, Chiti A, Sollini M. CNN-based approaches for PET/CT and PET/MR. Where do we stand?". Clin Transl Imaging. 2021;

18. Gozzi N. Explainable AI in medicine : application on EMG data for myo-controlled prosthesis. Italy; 2020.

19. Samek W, Montavon G, Vedaldi A, Hansen LK, Müller K-R, editors. Explainable AI: Interpreting, Explaining and Visualizing Deep Learning. Cham: Springer International Publishing; 2019.

20. Boellaard R, O’Doherty MJ, Weber WA, Mottaghy FM, Lonsdale MN, Stroobants SG, et al. FDG PET and PET/CT: EANM procedure guidelines for tumour PET imaging: version 1.0. Eur J Nucl Med Mol Imaging. Springer; 2010;37:181–200.

21. Boellaard R, Delgado-Bolton R, Oyen WJG, Giammarile F, Tatsch K, Eschner W, et al. FDG PET/CT: EANM procedure guidelines for tumour imaging: version 2.0. Eur J Nucl Med Mol Imaging. 2014;42:328–54.

22. Gallivanone F, Interlenghi M, Canervari C, Castiglioni I. A fully automatic, threshold-based segmentation method for the estimation of the Metabolic Tumor Volume from PET images: validation on 3D printed anthropomorphic oncological lesions. J Instrum. IOP Publishing; 2016;11:C01022–C01022.

23. Vezhnevets V, Konushin V. “GrowCut” - Interactive Multi-Label ND Image Segmentation By Cellular Automata. Graphicon. 2004;1.

24. Vallières M, Freeman CR, Skamene SR, El Naqa I. A radiomics model from joint FDG-PET and MRI texture features for the prediction of lung metastases in soft-tissue sarcomas of the extremities. Phys Med Biol. 2015;60:5471–96.

25. Stefano A, Gallivanone F., Grosso E., Russo G., Messa C., Tripoli V. GMC and CI. TOUCH-SUV: a Touchscreen-Assisted Tool for Quantitative, Reproducible, Clinically Feasible and Collaborative Diagnostic Reporting of Whole-Body PET-CT. Softw Eng. 2011;1:1–8.

26. Stefano A, Gallivanone F, Grosso E, Messa C, Gianolli L, Gilardi MC, et al. TOUCH-SUV: a Touchscreen-Assisted Tool for Quantitative, Reproducible, Clinically Feasible and Collaborative Diagnostic Reporting of Whole-Body PET-CT Studies. Int J Softw Eng. 2012;1:24–31.

27. Aerts HJ, Velazquez ER, Leijenaar RT, Parmar C, Grossmann P, Carvalho S, et al. Decoding tumour phenotype by noninvasive imaging using a quantitative radiomics approach. Nat Commun. 2014;5:4006.

28. Zwanenburg A, Leger S, Vallières M, Löck S. Image biomarker standardisation initiative [Internet]. [cited 2019 Oct 23]. Available from: http://arxiv.org/abs/1612.07003

29. Love MI, Huber W, Anders S. Moderated estimation of fold change and dispersion for RNA-seq data with DESeq2. Genome Biol. BioMed Central; 2014;15:550.

30. Rentzsch P, Witten D, Cooper GM, Shendure J, Kircher M. CADD: predicting the deleteriousness of variants throughout the human genome. Nucleic Acids Res. 2019;47:D886–94.

31. Molnar C. Interpretable Machine Learning. A Guide for Making Black Box Models Explainable. Book. 2019.

32. Carvalhal S, Ribeiro SA, Arocena M, Kasciukovic T, Temme A, Koehler K, et al. The nucleoporin ALADIN regulates Aurora A localization to ensure robust mitotic spindle formation. Zheng Y, editor. Mol Biol Cell. 2015;26:3424–38.

33. Zhang M-Y, Liu X-X, Li H, Li R, Liu X, Qu Y-Q. Elevated mRNA Levels of AURKA, CDC20 and TPX2 are associated with poor prognosis of smoking related lung adenocarcinoma using bioinformatics analysis. Int J Med Sci. 2018;15:1676–85.

34. Mehta A, Dobersch S, Romero-Olmedo AJ, Barreto G. Epigenetics in lung cancer diagnosis and therapy. Cancer Metastasis Rev. 2015;34:229–41.

35. Sui Q, Liang J, Hu Z, Chen Z, Bi G, Huang Y, et al. Genetic and microenvironmental differences in non-smoking lung adenocarcinoma patients compared with smoking patients. Transl Lung Cancer Res. Transl Lung Cancer Res; 2020;9:1407–21.

36. Airoldi I, Di Carlo E, Cocco C, Caci E, Cilli M, Sorrentino C, et al. IL-12 Can Target Human Lung Adenocarcinoma Cells and Normal Bronchial Epithelial Cells Surrounding Tumor Lesions. Ng IO-L, editor. PLoS One. 2009;4:e6119.

37. Liu Z, Yang W, Yang S, Cai K. The close association between IL‑12Rβ2 and p38MAPK, and higher expression in the early stages of NSCLC, indicates a good prognosis for survival. Mol Med Rep. 2018;

38. Kojima T, Shimazui T, Hinotsu S, Joraku A, Oikawa T, Kawai K, et al. Decreased expression of CXXC4 promotes a malignant phenotype in renal cell carcinoma by activating Wnt signaling. Oncogene. 2009;28:297–305.

39. Wissmann C, Wild PJ, Kaiser S, Roepcke S, Stoehr R, Woenckhaus M, et al. WIF1, a component of the Wnt pathway, is down-regulated in prostate, breast, lung, and bladder cancer. J Pathol. 2003;201:204–12.

40. Uematsu K, He B, You L, Xu Z, McCormick F, Jablons DM. Activation of the Wnt pathway in non small cell lung cancer: evidence of dishevelled overexpression. Oncogene. 2003;22:7218–21.

41. Zhao Y, Yang Z-Q, Wang Y, Miao Y, Liu Y, Dai S-D, et al. Dishevelled-1 and dishevelled-3 affect cell invasion mainly through canonical and noncanonical Wnt pathway, respectively, and associate with poor prognosis in nonsmall cell lung cancer. Mol Carcinog. 2010;n/a-n/a.

42. Lu H, Jin W, Sun J, Feng L, Lan H, Shen Q, et al. New tumor suppressor CXXC finger protein 4 inactivates mitogen activated protein kinase signaling. FEBS Lett. 2014;588:3322–6.

43. Li P, Luo X, Xie Y, Li P, Hu F, Chu J, et al. GC-Derived EVs Enriched with MicroRNA-675-3p Contribute to the MAPK/PD-L1-Mediated Tumor Immune Escape by Targeting CXXC4. Mol Ther Nucleic Acids. Mol Ther Nucleic Acids; 2020;22:615–26.

44. Stutvoet TS, Kol A, Vries EG, Bruyn M, Fehrmann RSN, Terwisscha van Scheltinga AGT, et al. MAPK pathway activity plays a key role in PD‐L1 expression of lung adenocarcinoma cells. J Pathol. 2019;249:52–64.

45. Zhang L, Chen J, Cheng T, Yang H, Li H, Pan C. Identification of the key genes and characterizations of Tumor Immune Microenvironment in Lung Adenocarcinoma (LUAD) and Lung Squamous Cell Carcinoma (LUSC). J Cancer. 2020;11:4965–79.

46. Law BYK, Michelangeli F, Qu YQ, Xu S-W, Han Y, Mok SWF, et al. Neferine induces autophagy-dependent cell death in apoptosis-resistant cancers via ryanodine receptor and Ca2+-dependent mechanism. Sci Rep. Sci Rep; 2019;9:20034.

47. Davis FM, Parsonage MT, Cabot PJ, Parat M-O, Thompson EW, Roberts-Thomson SJ, et al. Assessment of gene expression of intracellular calcium channels, pumps and exchangers with epidermal growth factor-induced epithelial-mesenchymal transition in a breast cancer cell line. Cancer Cell Int. 2013;13:76.

48. Vansteenkiste J, Crino L, Dooms C, Douillard JY, Faivre-Finn C, Lim E, et al. 2nd ESMO Consensus Conference on Lung Cancer: early-stage non-small-cell lung cancer consensus on diagnosis, treatment and follow-up. Ann Oncol. 2014;25:1462–74.

49. Wu W, Parmar C, Grossmann P, Quackenbush J, Lambin P, Bussink J, et al. Exploratory Study to Identify Radiomics Classifiers for Lung Cancer Histology. Front Oncol. 2016;6:1–11.

50. Koyasu S, Nishio M, Isoda H, Nakamoto Y, Togashi K. Usefulness of gradient tree boosting for predicting histological subtype and EGFR mutation status of non-small cell lung cancer on 18F FDG-PET/CT. Ann Nucl Med. 2019;

51. Kirienko M, Cozzi L, Rossi A, Voulaz E, Antunovic L, Fogliata A, et al. Ability of FDG PET and CT radiomics features to differentiate between primary and metastatic lung lesions. Eur J Nucl Med Mol Imaging. 2018;45:1649–60.

52. Groheux D, Quere G, Blanc E, Lemarignier C, Vercellino L, de Margerie-Mellon C, et al. FDG PET-CT for solitary pulmonary nodule and lung cancer: Literature review. Diagn Interv Imaging. Elsevier Masson; 2016;97:1003–17.

53. Nagano K, Maeda Y, Kanasaki S, Watanabe T, Yamashita T, Inoue M, et al. Ephrin receptor A10 is a promising drug target potentially useful for breast cancers including triple negative breast cancers. J Control Release. 2014;189:72–9.

54. Wang Y-T, Pan S-H, Tsai C-F, Kuo T-C, Hsu Y-L, Yen H-Y, et al. Phosphoproteomics Reveals HMGA1, a CK2 Substrate, as a Drug-Resistant Target in Non-Small Cell Lung Cancer. Sci Rep. 2017;7:44021.

55. Phan NN, Liu S, Wang C-Y, Hsu H-P, Lai M-D, Li C-Y, et al. Overexpressed gene signature of EPH receptor A/B family in cancer patients-comprehensive analyses from the public high-throughput database. Int J Clin Exp Pathol. 2020;

56. Bai H, Duan J, Li C, Xie W, Fang W, Xu Y, et al. EPHA mutation as a predictor of immunotherapeutic efficacy in lung adenocarcinoma. J Immunother Cancer. 2020;

57. Kirienko M, Cozzi L, Antunovic L, Lozza L, Fogliata A, Voulaz E, et al. Prediction of disease-free survival by the PET/CT radiomic signature in non-small cell lung cancer patients undergoing surgery. Eur J Nucl Med Mol Imaging. 2018;45:207–17.
